# Metabolomics-Based Study of Logarithmic and Stationary Phases of Promastigotes in *Leishmania major *by ^1^H NMR Spectroscopy

**DOI:** 10.7508/ibj.2016.02.002

**Published:** 2016-04

**Authors:** Mohammad Arjmand, Azadeh Madrakian, Ghader Khalili, Ali Najafi, Zahra Zamani, Ziba Akbari

**Affiliations:** 1 Dept. of Biochemistry, Pasteur Institute of Iran, Tehran, Iran;; 2 Dept. of Microbiology, Islamic Azad University, Pharmaceutical Sciences Branch, Tehran, Iran;; 3 Dept. of Immunology, Pasteur Institute of Iran, Tehran, Iran

**Keywords:** *Leishmania major*, Metabolomics, Principal component analysis

## Abstract

**Background:**

Cutaneous leishmaniasis is one of the most important parasitic diseases in humans. In this disease, one of the responsible organisms is *Leishmania major*, which is transmitted by sandfly vector. There are specific differences in biochemical profiles and metabolite pathways in logarithmic and stationary phases of Leishmania parasites. In the present study, ^1^H NMR spectroscopy was used to examine the metabolites outliers in the logarithmic and stationary phases of promastigotes in *L. major* to enlighten more about the transmission mechanism in metacyclogenesis of *L. major*.

**Methods:**

Promastigote was cultured, logarithmic and stationary phases were separated by the peanut agglutinin, and cell metabolites were extracted. ^1^H NMR spectroscopy was applied, and outliers were analyzed using principal component analysis.

**Results:**

The most altered metabolites in stationary and logarithmic phases were limited to citraconic acid, isopropylmalic acid, L-leucine, ornithine, caprylic acid, capric acid, and acetic acid.

**Conclusion:**

^1^H NMR spectroscopy could play an important role in the characterization of metabolites in biochemical pathways during a metacyclogenesis process. These metabolites and their pathways can help in exploiting a transmission mechanism in metacyclogenesis, and outcoming data might be used in the metabolic network reconstruction of *L. major *modeling.

## Introduction

Protozoan Leishmania spp. parasites involve about 20 species that infect humans by the bite of the sandfly vector^[^^1^^]^. Cutaneous leishmaniasis is the most popular form of the disease causing ulcers on skin^[^^2^^]^*. Leishmania major* and *L. tropica* are the main causes of this disorder found in Iran, Iraq, and their neighboring countries^[^^3^^,^^4^^]^. Leishmania cells exist in two morphological forms: free extracellular, flagellated promastigote in the gut of the sandfly vector, which changes into the intracellular, non-flagellated amastigote in the mammalian macrophage phagolysosome^[^^5^^,^^6^^]^. Parasites experience several developmental transitions during their infectious cycle. Procyclic promastigotes, similar to *in vitro* logarithmic promastigotes, are flagellated and motile forms that exist in the gut of sandfly. They divide rapidly but do not infect the vertebrate host. After developing into the metacyclic phase resembling the *in vitro* stationary phase, which is non-dividing and infective, promastigots are transferred to the sandfly's mouth. This metacyclogenesis process transforms from non-infective dividing procyclics to infective non-dividing metacyclics^[^^7^^-^^9^^]^.

*L. major *Fredlin was the first protozoan model that its metabolic network was reconstructed by Chavali *et al.*^[^^10^^]^ in 2008. The reconstructed metabolic network contained 560 gene spanning, 36 chromosomes of the genome, 1112 reaction, and 1047 metabolites^[^^10^^]^. Since there is a lack of complete information on *L*.* major* proteins and metabolites, the reconstruction was mostly based on protein expression from *L*. *infantum*, which is a related Leishmania spp^[^^11^^]^. In 2007, Leifso *et al.*^[^^11^^]^ reported that the stage-specific metabolism in amastigotes and promastigotes of *L. major*. They postulated that alcohol dehydrogenase enolase adenosine triphosphate synthase were preferentially expressed on promastigote stages while hexokinase was preferentially expressed in amastigotes. Alteration in enzyme level often functions as an intermediate step that leads to functionally relevant variation at the metabolite level^[^^12^^]^.

Metabolomics is a new field in post-genomic era and analyzes the changes of the whole metabolome simultaneously in response to environmental or cellular changes^[^^13^^]^. It is also a method for the high-throughput identification and quantification of metabolites in the metabolome of the cells, tissues, and organisms^[^^14^^]^. Proton NMR spectroscopy is a powerful, simple, reproducible, reliable, and quantitatively accurate tool that is typically applied to acquire metabolomics data^[^^15^^]^. Direct analysis of cellular metabolome is being applied to get more information about metabolic pathways and has been extensively used in many organisms^[^^16^^]^. There are several examples of its application in analyzing the metabolome of pathogenic microorganisms^[^^12^^]^. Therefore, to know the gradual changes in enzyme expression or drug inhibition of a special pathway, the full understanding of Leishmania metabolism, by detailed analysis of metabolic networks in different phases, is needed. There are specific differences in biochemical characters and metabolite pathways in the two phases of procyclic and metacyclic biochemical pathways of *L. major*^[^^17^^]^, which annually causes 20,000 infection cases in Iran^[^^18^^]^.

Thus, in the present pilot study, we examined the metabolites outliers of procyclic and metacyclic phases to better enlighten the transmission mechanism in metacyclogenesis of *L. major*. In addition, the out coming data might be used in the metabolic network reconstruction of *L. major* modeling. 

## MATERIALS AND METHODS

**Parasites**

The *L. major *promastigote (MRHO/IR/75/ER) was originally obtained from the infected BALB/c mice in Immunology Department of Pasture Institute of Iran (Tehran). The starting parasite inoculation was 2×10^6^/ml. Promastigotes were adapted at 23-25ºC in a Scheneider's medium (Invitrogen, Carlsbad, USA) supplemented with 10% inactivated fetal calf serum, 2 mM L-Glutamine, 100µg/ml streptomycin, and 100 U/ml penicillin (all from Sigma, CA, USA). The promastigotes were incubated with medium refreshment intervals for 15 days and then transferred into two flasks. Each flask contained a total volume of 80 ml with a cell density of approximately 14×10^6 ^viable cells/ml. Content of one flask was cultured for 5-7 days and represented as procyclic promastigote. The content of other flask was cultured from day15 to day 20 without adding fresh media, and then metacyclic promastigotes were harvested. In the current study, the purification of metacyclic promastigotes was accomplished by agglutination with peanut agglutinin (PNA; Sigma, CA, USA)^[^^19^^]^. In brief, PNA was added to washed metacyclic promastigotes to a final concentration of 50 µg/ml for 3×10^8^ parasite/ml. In the next step, PNA^-^ parasites (metacyclic promastigotes) were separated from agglutinated PNA^+^ parasites (procyclic promastigotes) by centrifugation at 200 ×g for 10 min. The non-agglutinated metacyclic promastigotes were then collected from the supernatant. Metacyclic and procyclic promastigotes were washed in PBS and collected by centrifugation at 3500 ×g at 4°C for 20 min. Then the increased infectivity of the PNA^-^metacyclic promastigotes was tested in 10 BALB/c mice (6–8 weeks old). The animals were selected randomly and divided into two groups: one group received subcutaneous injections of10^6 ^procyclic promastigotes, and the other group received 10^6 ^metacyclic promastigotes in the left hind footpad^[^^20^^,^^21^^]^. The footpad inflammation of the two groups were measured weekly, and their sizes were deﬁned as the mean of thickness and the width of footpad^[^^20^^,^^21^^]^. Ten samples, each containing 6×10^8^ promastigote/ml, were used for proton NMR spectroscopy from each phase.

**Cell extraction for metabolite analysis**

 In total, 6×10^8^ cell/mL were obtained from each vial, centrifuged at 1000 ×g at 4°C for 15 min and washed twice with ice-cold normal saline. Chilled perchloric acid (1.8 M) was added to the cell suspension, vortexed and sonicated at 4°C for 5 min, followed by centrifugation at 12000 ×g for 10 min at 4ºC. The pH of the supernatant was adjusted to 6.8, and then the supernatant was kept on ice for one hour to allow precipitation of potassium perchlorate and finally centrifuged again. The supernatant was lyophilized and taken for NMR spectroscopy^[^^22^^]^.

^1^ **HNMR experiments**

Lyophilized powder samples (n=10) from each phase were resuspended in D_2_O with 0.2 mM trimethylsilyl propionate (Sigma, CA, USA) as the NMR chemical shifts reference. Samples were centrifuged at 45,000 ×g at 4ºC for 20 min. The extracted samples were analyzed on a Bruker AV-500 NMR spectrometer with filed gradient operating at 500.13 MHz for proton observation at 298K. One-dimensional ^1^H NMR spectra were obtained using a 10-μs pulse, 6009.6 Hz spectral width, 0.1 s mixing time, 3.0 s relaxation delay, and 3000 transients with standard 1D NOESY (nuclear Overhauser spectroscopy) pulse sequence to suppress the residual water peak. 

**Data analysis**

The resulting NMR spectra were imported into MATLAB (v.7.8.0.347) software and preprocessed with ProMetab software (version 1.1). Chemical shifts between 0 and 10 ppm were taken and normalized. Spectra were binned in 0.004 units, and water peak (4.7 ppm) was removed for data analysis^[^^23^^]^. Principal component analysis, which is an unsupervised pattern recognition method that converts multidimensional data space into low dimensional model, was used. The spectral areas of above 6 ppm were removed. The outliers were identified, and the corresponding metabolite was extracted from metabolome database. Using MetaboAnalyst 2.0 (www.Metaboanalyst.ca), biochemical pathways were also investigated.

## RESULTS

**Fig. 1:**
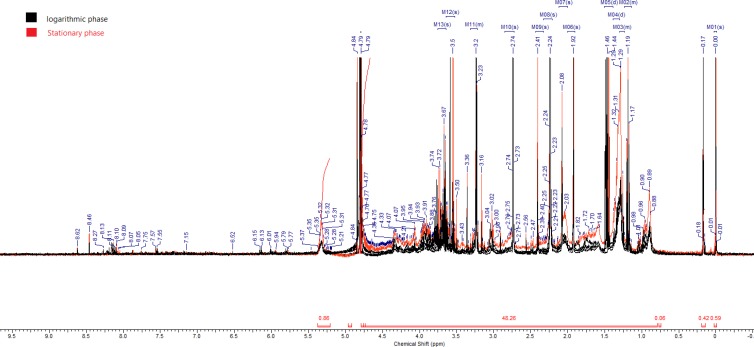
Superimposed 500 MHz H1NMR spectra of logarithmic and stationary phases of *L. major* ^1^H NMR. Metabolites were assigned to resonances within the aliphatic regions (-0.05-9.5 ppm) of the NMR spectra. Trimethylsilyl propionate was used as the internal standard for chemical shift calibration

Results from the present study showed that many metabolites pathways, influenced by Leishmania cell differentiations, were altered. The comparison of the metabolites patterns in stationary and logarithmic phases is depicted in Figure 1.

**Fig. 2:**
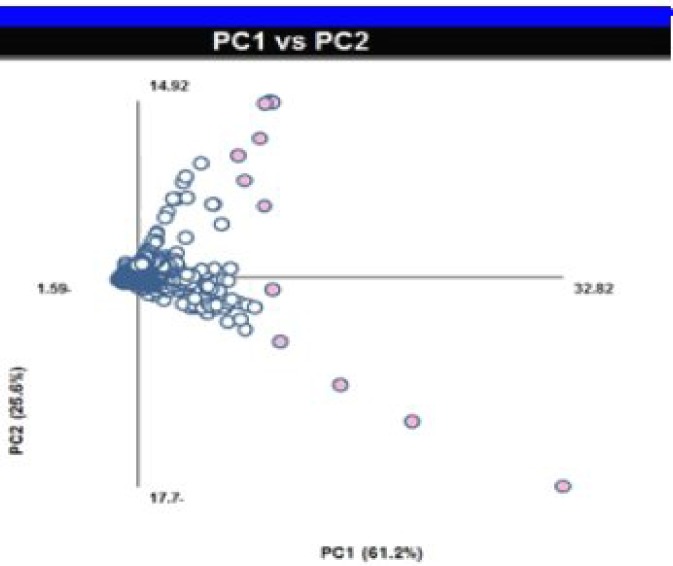
Loading plot (PCA_1_vs.PCA_2_). Axis was rotated for factor matching, and each circle shows specific binned chemical shift

**Fig. 3:**
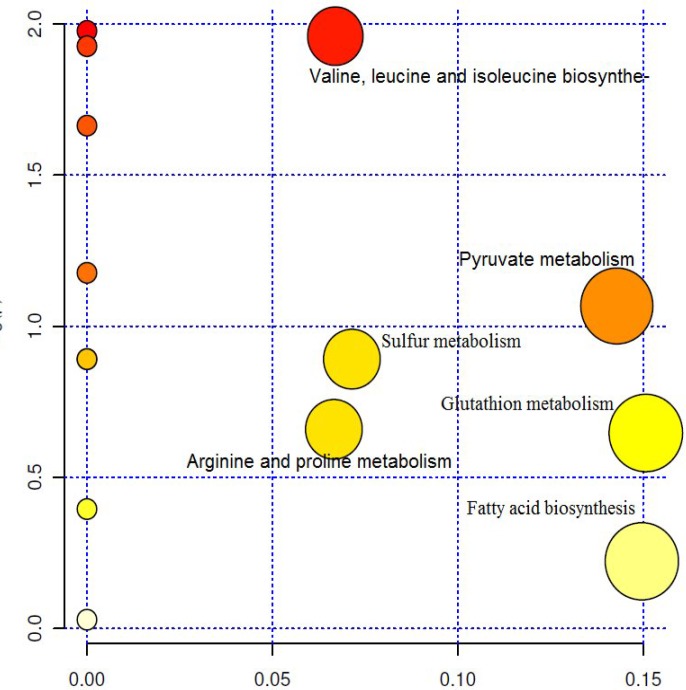
Summary of pathway analysis in stationary and logarithmic phases according to the degree of centrality

The PCA results (Table 1) showed that PCA1 and PCA2 contain 89.09% of data cumulations. Outliers were separated by the help of loading plots (Fig. 2). Metabolic pathways relating to these separated metabolites were studied using MetaboAnalyst 2.0 Database. Most altered metabolic pathways in stationary and logarithmic phases are shown in Table 2. Processing and analyzing the data using the above-mentioned database showed about 17 metabolic pathways that have one to three metabolites concentration changes in the logarithmic and stationary phases. Figure 3 also shows a summary of pathway analysis in these two phases, indicating the importance of pathways according to the degree of centrality. 

## DISCUSSION

*L. major* metabolism is remarkably complex and unique in their capability for polycistronic transcription^[^^24^^]^, the presence of kinetoplast DNA, glycosome, acidocalcisome, flagellar compartments^[^^25^^]^, and the occurrence of fatty acid synthesis mechanism. Chavali and coworkers^[^^10^^]^ reported that 12% of single-gene knockout in the *L. major* network are lethal and 10% are growth-reducing factors, which control the metabolism network of growth in parasites. Focusing on the metabolic cause of pathogenicity of *L. major* promastigotes, H^1^NMR spectroscopy of the logarithmic and stationary phases showed that there are 

**Table 1:** Principal component analysis

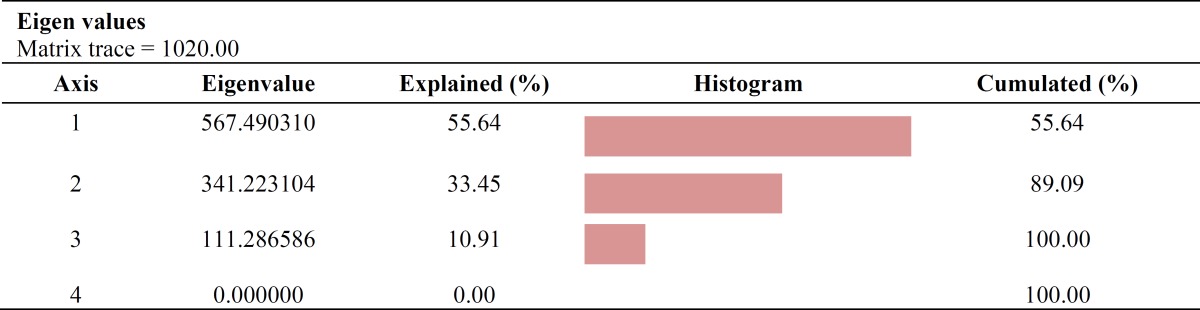

PCA1 (axis1) vs. PCA2 (axis2) contain 89% matrix information. PCA, principal component analysis

variations in some levels of individual metabolites. According to obtained *P *values (Table 2), Valine, leucine and isoleucine biosynthesis, pyruvate metabolism, glutathione metabolism, fatty acid biosynthesis, and sulfur metabolism are the most important pathways changed between these two phases. Leishmania parasites are auxotrophic for many amino acids and use the essential amino acids from their growth medium. Amino acids can also be used as major energy sources by Leishmania parasite^[^^26^^]^. It can be considered that amino acids play a key role in the Krebs cycle and eventually in NADH and ATP production. In the current study, the major alterations of amino acids in the procyclic and metacyclic phases of promastigotes are confined to three metabolites, named citraconic acid, isopropylmalic acid, and L-leuceine,in valine, leucine, and isoleucine biosynthesis pathway, respectively. The investigation of branched amino acids in leishmania by Ginger *et al*.^[^^27^^]^ revealed that the metabolism of these ketogenic amino acids prepare substrates for TCA cycle and energy supply forthe cells. Also, studies have indicated that the uptake rate of glucose and amino acids in the logarithmic phase is higher than the stationary phase of Leishmania^[^^28^^,^^29^^]^. Therefore, it is likely that in our investigation, the production of NADH during amino acid metabolism of valine, leucine, and isoleucine can partly satisfy the demand for energy source in the procyclic phase of promastigotes. Furthermore, these changes are due to the reduction in the catabolism level of enzymes. It has been reported that the rate of catabolism of amino acids by washed cells is decreased by increasing culture age, through reduction in the catabolism of enzyme level^[^^30^^]^.

The Leishmania parasite is motile and divides rapidly in the logarithmic phase^[^^31^^]^; therefore, it is delineated that this phase is more energy dependent than the stationary phase. Also, according to the result of Louassini *et al.*^[^^32^^]^, in the logarithmic phase, the parasite consumes an important quantity of glucose throughout glycolysis, with pyruvate as the end product. Thus, regarding the increase of glucose consumption in the logarithmic phase^[^^28^^]^, it can be concluded that pyruvate metabolism pathway of this phase is more active than the stationary phase. Our results showed alteration in isopropylmalic acid and acetate in pyruvate metabolism. Tracking of pyruvate metabolism in KEGG pathway database indicated that isopropylmalic acid, which is produced from acetyl-CoA, can change to acetate. Castilla *et al.*^[^^33^^]^ demonstrated that in high-glucose media, both promastigotes and amastigotes consume glucose and oxidize it to succinate, acetate, alanine, and CO_2_. In addition, Blum^[28]^ and Castilla *et al.*^[^^33^^]^ demonstrated that the alteration in the acetate is due to the increase in the consumption of glucose in the logarithmic phase, which is in consistent with our results.

Ornithine is a central part of the urea cycle for the disposal of excess nitrogen derived from amino acids together with ascorbic acid, and can act as the intermediate metabolite of the glutathione pathway. Our findings showed that glutathione profile alteration is confined to two metabolites, ornithine and ascorbic acid. It is also of interest that *L. major* uses ascorbic acid in defense against oxidative stress^[^^34^^]^. In the current study, the apparent alteration in two metabolites might be the result of high demand for amino acid as a source of energy and also due to the increase of oxidative stress in the active state of the logarithmic phase.

**Table 2:** Results from the pathway analysis in stationary and logarithmic phases

**Metabolic pathway**	**Metabolite**	**Total**	**Hits**	**Raw p**	**FDR**
Valine, leucine, and isoleucine biosynthesis	Citraconic acid, isopropylmalic acid, L-leucine	27	3	0.000068	0.005
Pyruvate metabolism	Acetic acid, isopropanol	32	2	0.004560	0.171
Glutathione metabolism	Ascorbic acid, ornithine	38	2	0.006400	0.171
Fatty acid biosynthesis	Caprylic acid , capric acid	49	2	0.010500	0.210
Sulfur metabolism	Acetic acid	18	1	0.058400	0.709

Total, the total number of compounds in the pathway; Hits, the actually matched number from the user uploaded data; Raw p, the original *P* value calculated from the enrichment analysis; FDR (false discovery rate), the *P* value adjusted using FDR

As promastigotes age and/or transform into amastigotes^[^^35^^]^, fatty acids serve as highly important energy sources and the rate of oxidation of fatty acids by *L. major* promastigotes increases with culture age^[^^28^^]^. Denny and Smith^[^^36^^]^ have confirmed that differentiation from procyclic to metacyclic promastigotes of leishmania can involve changes in membrane lipid composition. There are alterations in the distribution of lipophosphoglycan in to lipid rafts. Silva *et al.*^[^^37^^]^ study revealed that the metacyclic Phase in comparison with the procyclic phase of promastigotes has lower levels of unsaturated fatty acids, which is decreased in membrane fluidity in this stage. In the present study, caprylic and capric acids in fatty acid biosynthesis have shown changes between two phases of promastigotes. These two acids are considered as medium-chain saturated fatty acids and account for 65% and 35% of the composition of medium-chain triglycerides^[^^38^^]^, which are the sources of abundant and rapidly available energy in the leishmania parasite^[^^28^^]^. Blum *et al.*^[^^28^^]^investigated the rate of oxidation of short, medium, and long-chain fatty acids by stationary and logarithmic phases of *L. major*. Their results disclosed that the stationary phase promastigotes oxidized some saturated fatty acids (such as medium-chain fatty acids) three-four-fold faster than the logarithmic phase in the medium to induce oxidative phosphorylation in glycosomes and mitochondria and finally gain energy in these stages. Therefore, it can be postulated that caprylic acid and capric acid have an important role in the stationary phase of *L. major* metabolic network, and our results are in consistent with those data.

The current study indicates the alteration of acetate metabolite in the sulfur metabolism pathway as well. Some amino acids contain different levels of sulfur that finally could degrade to acetate by a simple hydrolysis of the thioester bond^[^^39^^]^. In *Trypanosome brucei*, acetate, which is produced from glucose and threonine amino acid in the mitochondrion, is synthetically essential to feed the necessary fatty acid biosynthesis and ATP production through the acetate shuttle. This shuttle has been recently described in the procyclic form of the parasite in insect^[^^40^^]^. Therefore, the utilization of high glucose, which is correlated with acetate production in this pathway and the presence of acetate shuttle in the procyclic phases, could be considered as a factor that makes acetate to be altered more in the logarithmic phase than stationary phase of promastigotes.

Overall, our findings show how the application of metabonomics approaches can play an important role in the characterization of biochemical pathways in different stages of promastigotes by identifying metabolites outliers in infective and non-infective forms of the promastigote in *L. major*. It could be concluded that the main sources of the energy backbone are affected by promastigotes differentiations in the metacyclogenesis process, and these metabolic outliers could be used in coming *in silico* metabolic modeling and gene knockout.
